# Evaluation of Pharmaceutical Compounding Training in the Australian Undergraduate Pharmacy Curricula

**DOI:** 10.3390/pharmacy8010027

**Published:** 2020-02-26

**Authors:** Sam Kosari, Vera H. Buss, Gregory M. Peterson, Kwang Choon Yee, Mark Naunton, Mary Bushell, Leroy Chiu, Jackson Thomas

**Affiliations:** 1 Discipline of Pharmacy, Faculty of Health, University of Canberra, Bruce, ACT 2617, Australia; Kwang_Choon.Yee@canberra.edu.au (K.C.Y.); mark.naunton@canberra.edu.au (M.N.); Mary.bushell@canberra.edu.au (M.B.); leroykschiu@gmail.com (L.C.); Jackson.thomas@canberra.edu.au (J.T.); 2 Centre for Primary Health Care and Equity, University of New South Wales, Randwick, NSW 2052, Australia; v.buss@student.unsw.edu.au; 3 Faculty of Health, University of Tasmania, Hobart 7005, Tasmania, Australia; g.peterson@utas.edu.au

**Keywords:** pharmacy education, pharmacy curriculum, extemporaneous dispensing, compounding

## Abstract

**Introduction:** In recent decades the role of the Australian community pharmacist has evolved to focus primarily on pharmaceutical care provision. Despite this, compounding remains an important product service offered by pharmacists. The aim of this study was to qualitatively describe the current integration of training in compounding within Bachelor of Pharmacy courses in Australia. **Methods:** The Australian Health Practitioner Regulatory Agency website was searched to identify eligible university courses. Subsequently, the educational providers’ homepages were consulted, and Bachelor of Pharmacy handbooks and curricula perused. All relevant information regarding training in compounding was extracted. **Results:** In total, 16 Bachelor of Pharmacy courses were identified. All of these contain compounding training in their curricula, including laboratory classes. Most curricula have units specifically dedicated to compounding and drug formulation. Three universities offer a curriculum which is organ-systems based, and include compounding relevant to the individual organ systems. **Discussion and Conclusions:** In Australia, the training in compounding is well integrated into pharmacy curriculum and is more emphasised than in many other developed countries. This is congruent with the International Pharmaceutical Federation’s needs-based approach to local pharmacy education. In Australia there is a need for pharmacists to routinely dispense simple compounded products. Further research is required to evaluate Australian pharmacy graduates’ compounding abilities and how best to promote the achievement of the required knowledge and skills to enable simple compounding.

## 1. Introduction

The role of pharmacists has greatly changed in the last 50 years. The new focus on the safe and effective use of medicines highly differs from the traditional role of pharmacists that was mainly centered on manufacturing and dispensing medication [1]. In Australia, the pharmacy curricula have been progressively adjusted to address the new challenges that pharmacists are facing [2]. Nevertheless, it should be acknowledged that pharmacies are still offering compounding services which require the pharmacists to be also equipped with this set of skills [3,4]. 

Compounding is defined as the preparation and supply of a single ‘unit of issue’ of a therapeutic product intended for supply for a specific patient [5]. The Pharmacy Board of Australia (PBA) sets out *Guidelines on Compounding of Medicines* for registered pharmacists and those who are in the process of becoming registered [5]. These guidelines should be read in accordance with relevant legislation and practice standards, such as the *Therapeutic Goods Act 1989* and standards published by the Pharmaceutical Society of Australia [5]. Medicines should only be compounded if there is no commercial product available, or the available product is not suitable, or if it is intended for research purposes. The compounding procedure is divided into two levels of difficulty — simple and complex [5]. Every registered pharmacist has the competency to conduct simple compounding, which is defined as “*the preparation and supply of a single ‘unit of issue’ of a therapeutic product intended for supply for a specific patient in response to an identified need*” [5]. For students to pass pharmaceutical units, their competency to prepare simple compounded products is assessed. Simple compounding routinely involves the compounding of products from formulations published in reputable references such as the *Australian Pharmaceutical Formulary and Handbook* (excluding the preparation of sterile products, which is considered complex compounding), or using other formulations for which information confirming quality, stability, safety, efficacy and rationality is available [5]. Examples of simple compounding include the preparation of topical creams, ointments, lotions, gels, oral liquids (mixtures, elixirs, solutions, suspensions, emulsions,) tinctures, powders, capsules, suppositories, pessaries. Complex compounding is considered beyond the scope of the standard pharmacy curriculum and requires the pharmacist to adequately expand their scope of practice [5]. Examples of complex compounding includes the preparation of parenterals, cytotoxics, modified-release dosage forms and ophthalmic preparations.

The main pathway to study pharmacy in Australia, is a four-year bachelor’s degree (and a less common pathway is a postgraduate two-year Master of Pharmacy). The Australian Pharmacy Council (APC) is responsible for the accreditation of education providers, including their proposed curricula, as outlined in the Standards for Pharmacy Programs in Australia and New Zealand. Any significant changes to the curriculum need to be approved by the APC [6]. In the appendix of the APC Standards for Pharmacy Programs, the six pharmacy learning domains are explained; one of the six domains, is *“Medicines: the medicinal product. The formulation and compounding of medicines, taking the pure drug substance and producing a dosage form for administration to the consumer, are at the heart of pharmaceutical science”* [6]. Therefore, this domain should be part of each pharmacy curriculum in Australia [6]. The aim of this study was to qualitatively analyse the integration of compounding within the curricula of all Australian Bachelor of Pharmacy courses.

## 2. Methods 

First, all eligible pharmacy courses were identified based on pre-defined inclusion criteria (approved programs of study according to the Australian Health Practitioner Regulatory Agency (AHPRA), bachelor’s degree, active in 2018, offered in Australia). The AHPRA homepage was searched [7]. Subsequently, the homepages of the universities offering Bachelor of Pharmacy programs in Australia were consulted to inspect the study handbook and the curriculum. The individual units were scanned for relevant content regarding compounding. The focus was on units that included practical training for compounding conducted in community pharmacies (simple compounding); units that solely dealt with advanced drug formulation techniques that are applied in the pharmaceutical industry were not considered for the analysis. Relevant information regarding the units was extracted (unit description and aim, pre-requirements). A second reviewer checked the first reviewer’s abstraction to ensure it was complete and accurate. It was also considered whether the course was taught in semesters, or trimesters, and whether students were based on campus or if the program was taught online. The data was extracted in a spreadsheet using Microsoft Excel 2016 (Microsoft Co, Washington, USA). For this project, ethics approval was not required since the data used is fully available online. 

## 3. Results

In total, 16 Bachelor of Pharmacy courses were identified which fulfilled the criteria (Table 1). These courses were offered by 16 different education providers in Australian (not including offshore locations). Two universities offered their courses in trimesters, and the remaining had a semester-structure. All pharmacy courses comprised units dealing with compounding. These units were not offered in the first semester or the last year of study; usually they were offered in the second and/or third year of study when students have already gained some basic knowledge of science and pharmacy. The universities offered the drug formulation units with increasing complexity; starting with simple compounding and continuing with more challenging drug formulations. At all universities, the students needed to successfully complete other units prior to commencing (prerequisites) or simultaneously (corequisites) with the compounding units. In all courses, laboratory classes were integrated in the units and taught in face-to-face sessions to ensure that the students learn the theoretical as well as the practical skills required to compound extemporaneous medicines. In general, all universities offered the pharmacy course on campus, except that the University of New England offered an additional online course. In this case, the students needed to visit mandatory intensive schools for some units, including the compounding unit. 

Besides these similarities among the courses, there was one noteworthy difference — the way the compounding units were implemented in the curriculum. Most universities offered specific units that provided students with the essential skills, usually held in the second year and split over several semesters. These units had names such as Dosage Form Design or Pharmaceutical Formulation. An example description for such a unit is How Medicines Work III at Monash University:

*“This unit will provide students with a detailed understanding of the specific biopharmaceutical and formulation considerations for various drug delivery routes […] This unit will also address specific issues to be considered when formulating and dispensing medicines and the differences in quality, activity and toxicity that may arise from variability in manufacturing and formulation processes, with a focus on Good Manufacturing Practice”.*

[24]

There were three universities that have a different approach: the James Cook University, the Queensland University of Technology, and the University of Newcastle did not offer specific compounding units; instead, their units were based on integrated organ-systems. This means that the units each focused on individual organ systems; within the units, one teaching aspect is drug formulation specific to the treatment of medical conditions relating to this organ system. For example, at the James Cook University one unit in the pharmacy curriculum is called Dermatology for Pharmacists; it focusses on all aspects of skin conditions from the physiology to compounding of topical preparations. The following is an excerpt of the unit description:

*“An emphasis will be placed on the care of the skin, identification of common skin conditions, patient education and prevention. Students will also explore the pharmaceutics of formulations and dosage forms relevant to dermatology and apply these skills in relation to creams, gels, lotions, liposomal preparations, paints and tinctures”.*

[25]

## 4. Discussion

The aim of this study was to investigate the integration of compounding training in the pharmacy curriculum in Australia. Based on the online published curricula, all Australian universities have integrated compounding in their Bachelor courses. All universities developed an approach for their curricula that builds on previously acquired knowledge and skills to reinforce the learned content (prerequisites). Additionally, the curricula often present related content in the same semester (corequisites). This approach can be called spiral integration; according to Harden and Stamper, there are four important features in spiral curricula: (1) topics are revisited, (2) increasing levels of difficulty, (3) new learning is related to previous, and (4) competence increases [26]. These features could be observed in the reviewed pharmacy curricula relevant to compounding. Also, all universities directly included laboratory classes in the units to combine theory and practice. The advantage of this was confirmed through research showing that the students’ performance and retention were increased if lectures and laboratory classes were taught in the same semester. Furthermore, a study demonstrated that students benefitted from units integrating lectures and laboratory classes concomitantly [27]. A concept that has been introduced into some pharmacy curricula, and is especially popular in medical schools, is integrated teaching based on organ-systems [28]. Currently, there is some controversy around the topic of integrated pharmacy curricula, since there is no strong evidence that shows the benefits or superiority of such organ systems-based curricula over the traditional curricula [28,29].

Researchers from the University of Queensland analysed their pharmacy curriculum under the light of students’ engagement and shaping of future pharmacists [30]. They concluded that the examined curriculum focused more on skills and knowledge, than on the development of becoming a pharmacist [30]. By fragmenting the curriculum into key concept clusters, including one for pharmaceutics and formulation, the researchers perceived a danger of students never truly experiencing pharmacy practice during their studies, limiting their chance of shaping who they professionally are going to become [30]. Another study from the University of Queensland examined pharmacists’ and pharmacy students’ views of compounding. The authors raised some concerns in relation to the curriculum, such as a lack of transforming the learned content into practice, differences between the equipment used in the university laboratory compared to the community pharmacy, and a decreased confidence of final-year students due to a time gap since their last compounding class. Furthermore, the authors explained that after the findings of this study they had revised their curriculum in regard to compounding training [4]. The obstacles observed by the research team can be tackled during the internship year which is a compulsory part of becoming a registered pharmacist. During that time pharmacy interns should be given the opportunity to translate their university skills into practice under the supervision of an experienced pharmacist. 

In an international context, compounding is integrated into the curriculum of pharmacy schools globally [31,32]. However, recent research identified that compounding played a greater role for pharmacists in Australia than in other countries. This may be because In Australia the pharmacist is responsible for the direct preparation of extemporaneously compounded products. While it is common for intern pharmacists to prepare products under the direct supervision of the registered pharmacist, other pharmacy staff (pharmacy assistants, dispense technicians) in Australia do not compound products.

Researchers compared the Pharmacy Learning Outcome Frameworks of the United States of America, the United Kingdom, Canada, and Australia; the most significant difference observed was in regard to compounding, which is only emphasised in the Australian Framework [33]. The authors’ explanation for this observation was differences in jurisdictions regarding the competencies of pharmacy technicians [33]. In Australia, although students, interns, dispensary assistants or dispensary technicians can participate in compounding activities, it is the pharmacist’s responsibility to ensure that these individuals are sufficiently trained and work under the pharmacist’s direct supervision [5].

A recent commentary article authored by academics in the Netherlands has called for a revival of education in pharmacy schools on topics that underpin pharmaceutics. Conceding that in some developed countries curricula has removed too much content that enables knowledge and skill competency in the areas of drug formulation and pharmaceutics [34]. The International Pharmaceutical Federation (FIP) supports a needs-based approach to guide pharmacy education [35]. The FIP recognises a universal approach such as a global competency framework using a single global curriculum, across both developing and developed countries is not suitable. When reviewing international pharmacy school curriculum and evaluating incorporated pharmaceutical compounding training, it is important to recognise local population health needs [36].

## 5. Limitations

This study only serves the purpose of providing an overview of the way compounding was integrated in the pharmacy curriculum. The information used in the analysis is available online on the educators’ homepages; based on this, a direct comparison of the share of compounding in the different curricula is not possible since many universities offer units which comprise compounding along with other learning content. Therefore, further studies are required to obtain a deeper insight into the significance of compounding in the curriculum as well as into the Bachelor of Pharmacy graduates’ compounding abilities in Australia.

## 6. Conclusions

While examining the presence of compounding training in Australian pharmacy curricula, many similarities between the universities were observed. The compounding units were integrated somewhere in the middle of the courses and contained compulsory laboratory classes. Students were first introduced to simple compounding, followed by more complex formulations. One main difference that could be detected is the organ systems-based approach used by three universities. These educational providers did not offer specific compounding units; instead, the formulation training was integrated in units that were concerned with specific organ systems. Currently, there is not sufficient evidence to show superiority of one of the two approaches.

## Figures and Tables

**Table 1:** Overview of Bachelor of Pharmacy courses in Australia.

Educational Provider	Location	Mode	Structure
Charles Darwin University [8]	Casuarina, Northern Territory	On campus	Semesters
Charles Sturt University [9]	Orange, New South Wales	On campus	Semesters
Curtin University [10]	Bentley, Western Australia	On campus	Semesters
Griffith University [11]	Gold Coast, Queensland	On campus	Trimesters
James Cook University [12]	Townsville, Queensland	On campus	Semesters
La Trobe University [13]	Bendigo, Victoria	On campus	Semesters
Monash University [14]	Parkville, Victoria	On campus	Semesters
Queensland University of Technology [15]	Gardens Point, Queensland	On campus	Semesters
Royal Melbourne Institute of Technology [16]	Bundoora, Victoria	On campus	Semesters
University of Canberra [17]	Bruce, Australian Capital Territory	On campus	Semesters
University of New England [18]	Armidale, New South Wales	On campus or online	Trimesters
University of Newcastle [19]	Callaghan, New South Wales	On campus	Semesters
University of Queensland [20]	Wolloongabba, Queensland	On campus	Semesters
University of South Australia [21]	Adelaide, South Australia	On campus	Semesters
University of Sydney [22]	Camperdown, New South Wales	On campus	Semesters
University of Tasmania [23]	Hobart, Tasmania	On campus	Semesters
